# Cytoprotective effect of genistein against dexamethasone-induced pancreatic β-cell apoptosis

**DOI:** 10.1038/s41598-022-17372-z

**Published:** 2022-07-28

**Authors:** Kanchana Suksri, Namoiy Semprasert, Thawornchai Limjindaporn, Pa-thai Yenchitsomanus, Sirirat Kooptiwoot, Suwattanee Kooptiwut

**Affiliations:** 1 Division of Endocrinology, Department of Physiology, Faculty of Medicine Siriraj Hospital, Mahidol University, 2 Wanglang Road, Bangkoknoi, Bangkok, 10700 Thailand; 2 Department of Anatomy, Faculty of Medicine Siriraj Hospital, Mahidol University, Bangkok, Thailand; 3 Division of Molecular Medicine, Research Department, Faculty of Medicine Siriraj Hospital, Mahidol University, Bangkok, Thailand; 4 Department of Psychiatry, Faculty of Medicine Siriraj Hospital, Mahidol University, Bangkok, Thailand

**Keywords:** Molecular biology, Endocrinology

## Abstract

Steroid-induced diabetes is a well-known metabolic side effect of long-term use of glucocorticoid (GC). Our group recently demonstrated dexamethasone-induced pancreatic β-cell apoptosis via upregulation of TRAIL and TRAIL death receptor (DR5). Genistein protects against pancreatic β-cell apoptosis induced by toxic agents. This study aimed to investigate the cytoprotective effect of genistein against dexamethasone-induced pancreatic β-cell apoptosis in cultured rat insulinoma (INS-1) cell line and in isolated mouse islets. In the absence of genistein, dexamethasone-induced pancreatic β-cell apoptosis was associated with upregulation of TRAIL, DR5, and superoxide production, but downregulation of TRAIL decoy receptor (DcR1). Dexamethasone also activated the expression of extrinsic and intrinsic apoptotic proteins, including Bax, NF-κB, caspase-8, and caspase-3, but suppressed the expression of the anti-apoptotic Bcl-2 protein. Combination treatment with dexamethasone and genistein protected against pancreatic β-cell apoptosis, and reduced the effects of dexamethasone on the expressions of TRAIL, DR5, DcR1, superoxide production, Bax, Bcl-2, NF-κB, caspase-8, and caspase-3. Moreover, combination treatment with dexamethasone and genistein reduced the expressions of TRAIL and DR5 in isolated mouse islets. The results of this study demonstrate the cytoprotective effect of genistein against dexamethasone-induced pancreatic β-cell apoptosis in both cell line and islets via reduced TRAIL and DR5 protein expression.

## Introduction

Glucocorticoid (GC) drugs are known to have anti-inflammatory and immunosuppressive effects. Glucocorticoids are, therefore, widely used to treat several inflammatory and immune diseases, such as rheumatoid arthritis, asthma, and allergies^1^. GC drugs can induce several side effects such as osteoporosis, psychosis and diabetes^2,3^. A well-established metabolic side effect of long-term GC administration is steroid-induced diabetes^4–6^, and it has long been known that GCs induce insulin resistance and pancreatic β-cell apoptosis^7–9^.

The effects of GCs on pancreatic β-cells have been widely investigated since impairment of pancreatic β-cell function plays a crucial role in the development of diabetes. Several pathways that may induce pancreatic β-cell apoptosis have been proposed. First, GCs release heat shock protein (HSP) 90 from the glucocorticoid receptor (GR), and free Hsp90 activates calcineurin^10^. Stimulated calcineurin dephosphorylates the Bcl-2-associated death promoter (BAD), which separates BAD from the cytoplasmic adapter protein (CAP). BAD then moves to the mitochondrial membrane and forms a mitochondrial permeability transition pore (MPTP), thereby releasing cytochrome c from mitochondria to trigger apoptotic cascades^10^. Second, GCs increase intracellular oxidative stress by downregulating the antioxidant enzymes glutathione peroxidase-4 (GPx4)^11^, superoxide dismutase (SOD)^12^, and thioredoxin (Trx)^13^, but by upregulating the oxidase enzymes NADPH oxidase 4 (NOX4)^12^ and the thioredoxin interacting protein (TXNIP)^13^. This leads to accumulation of oxidative stress in pancreatic β-cells resulting in apoptosis. Third, GCs induce pancreatic β-cell apoptosis via endoplasmic reticulum (ER) stress. GCs activate the unfolded protein response (UPR) sensor inositol-requiring enzyme (IRE1), and activate transcription factor 6 (ATF6)^14^, which subsequently stimulates the transcription factor CCAAT/enhancer-binding protein homologous protein (CHOP). CHOP then upregulates pro-apoptotic proteins, including Bcl-2-associated X (Bax), Bcl-2 homologous antagonist-killer (Bak), Bcl-2-like protein 11 (Bim), and death receptor 5 (DR5)^15–17^. The upregulation of these pro-apoptotic proteins induces pancreatic β-cell apoptosis. Finally, GCs induce pancreatic β-cell apoptosis via mitogen inducible gene 6 (Mig6), which inhibits pancreatic β-cell proliferation by reduction of extracellular-signaling-related kinase (ERK) 1/2 phosphorylation. Furthermore, Mig6 directly blocks the G1/S transition phase of the cell cycle^18^. GCs reduce pancreatic β-cell mass by inducing pancreatic β-cell apoptosis and inhibiting pancreatic β-cell proliferation. Our group recently demonstrated that dexamethasone induces pancreatic β-cell apoptosis via upregulation of TRAIL and DR5 protein expression^19^.

Genistein has been studied both in vitro and in vivo for its protective effect against toxic substances that induce pancreatic β-cell apoptosis^20–23^. Genistein exerts its effects via multiple mechanisms and cell signaling pathways. For example, genistein prevents cytokine-induced pancreatic β-cell dysfunction via suppression of the NF-κB, ERK-1/2, and JAK/STAT pathways^22^. Genistein also reduces inflammation-induced diabetes by decreasing TNF-β secretion via activation of the ERK and p38 MAPKs-dependent pathways^24^. Moreover, genistein can stimulate pancreatic β-cell proliferation^20^. However, the protective effect of genistein against dexamethasone-induced pancreatic β-cell apoptosis is unknown. We hypothesized that genistein protects dexamethasone-induced pancreatic β-cell apoptosis via downregulation of TRAIL and DR5 protein expression, and via inhibition of the TRAIL apoptotic pathway. The aim of this study was to investigate the cytoprotective effect of genistein on dexamethasone-induced pancreatic β-cell apoptosis, TRAIL and DR5 protein expression, and the TRAIL apoptotic pathway in cultured rat insulinoma (INS-1) cell line and isolated mouse islets.

## Results

### Genistein protected against dexamethasone-induced apoptosis of INS-1 cells

**Figure 1:**
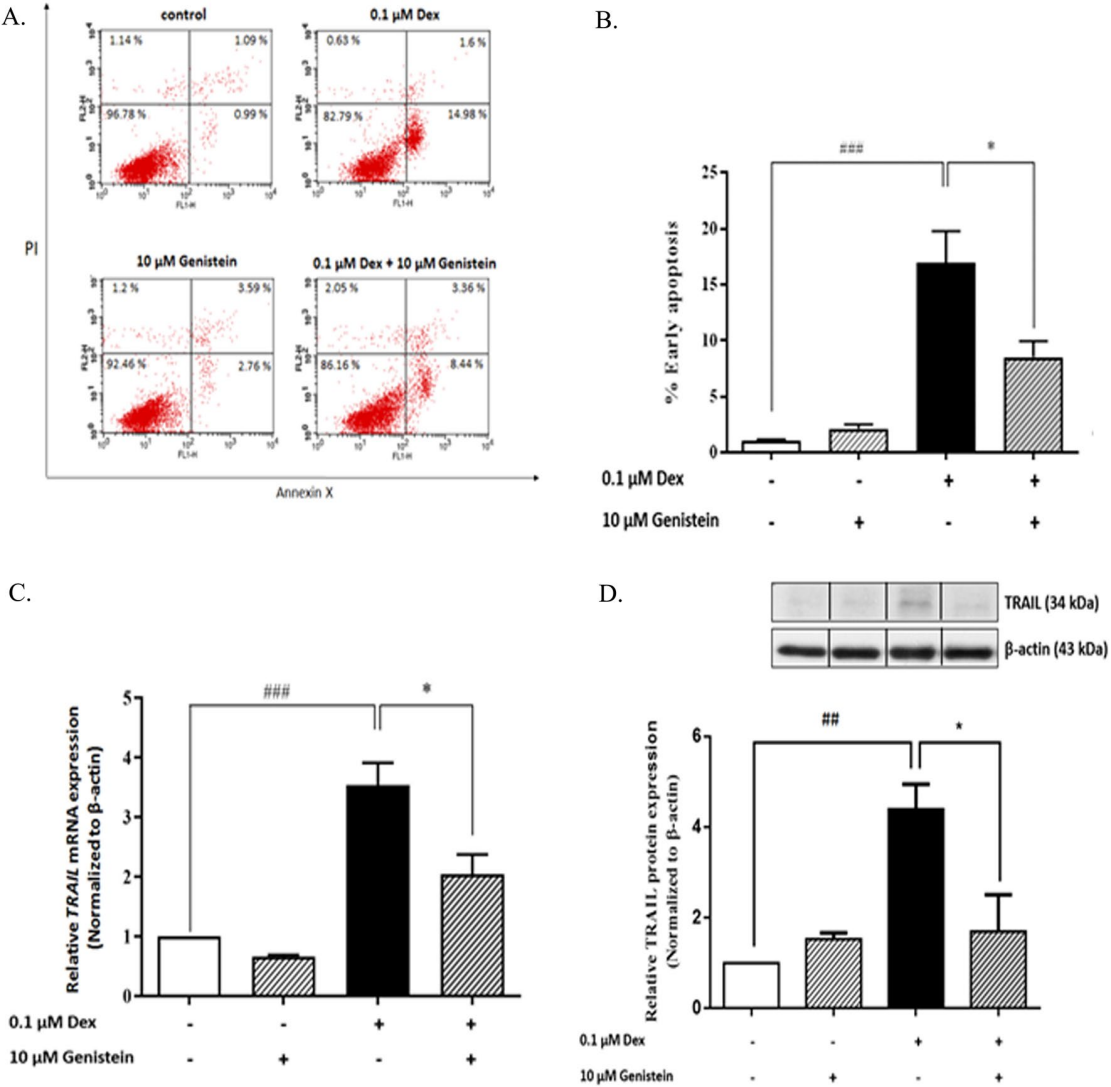
Effect of genistein on dexamethasone-induced pancreatic β-cell apoptosis and TRAIL expression. (**A**) A representative of AnnexinV/PI dot plot of the effect of dexamethasone and 10 μM genistein on percent apoptosis in INS-1 cells at 72 h. (**B**) The percentage of early apoptosis was measured by Annexin V/PI staining. (**C**) Fold change of TRAIL mRNA normalized to β-actin mRNA at 48 h. (**D**) A representative Western blot analysis of TRAIL and β-actin from INS-1 cells. All bands were cropped and full-length blots are shown in supplementary Fig. 1. The bar graph below is the TRAIL protein level normalized to β-actin protein. Data are expressed as mean ± SEM of 3 independent experiments. ###, *P* < 0.001 versus control, #, *P* < 0.01 versus control; *, P < 0.05 versus 0.1 µM dexamethasone.

To examine the effects of dexamethasone and genistein on pancreatic β-cells, INS-1 cells were cultured with either 0.1 µM of dexamethasone, 10 μM of genistein, or the combination of the two. Dexamethasone alone induced apoptosis of INS-1 cells, whereas combination dexamethasone and genistein significantly decreased apoptosis of INS-1 cells (Fig. 1A,B). Genistein treatment alone had no effect on INS-1 cells compared to control. These results indicate that 10 μM genistein significantly inhibits dexamethasone-induced pancreatic β-cell apoptosis in INS-1 cells.

### Genistein reduced dexamethasone-induced TRAIL mRNA expression in INS-1 cells

To examine the protective effect of genistein on dexamethasone-induced pancreatic β-cell apoptosis associated with TRAIL expression, INS-1 cells were cultured with 0.1 μM dexamethasone with or without 10 μM genistein. At 48 h after treatment, expression levels of TRAIL mRNA and protein in INS-1 cell lysate were examined by real-time RT-qPCR and Western blot analysis, respectively. Relative TRAIL mRNA expression is shown in Fig. 1C. INS-1 cells cultured with dexamethasone alone significantly upregulated TRAIL mRNA expression. However, when they were co-cultured with the combination of dexamethasone and genistein, TRAIL mRNA expression was significantly decreased compared to control. Similarly, INS-1 cells cultured with dexamethasone alone significantly increased TRAIL protein expression compared to control. Combination treatment with dexamethasone and genistein significantly reduced TRAIL protein expression compared to that observed in dexamethasone treatment alone (Fig. 1D). Genistein treatment alone had no significant effect on TRAIL mRNA or protein expression compared to control.

### Genistein reduced dexamethasone-induced TRAIL expression in isolated mouse islets

**Figure 2:**
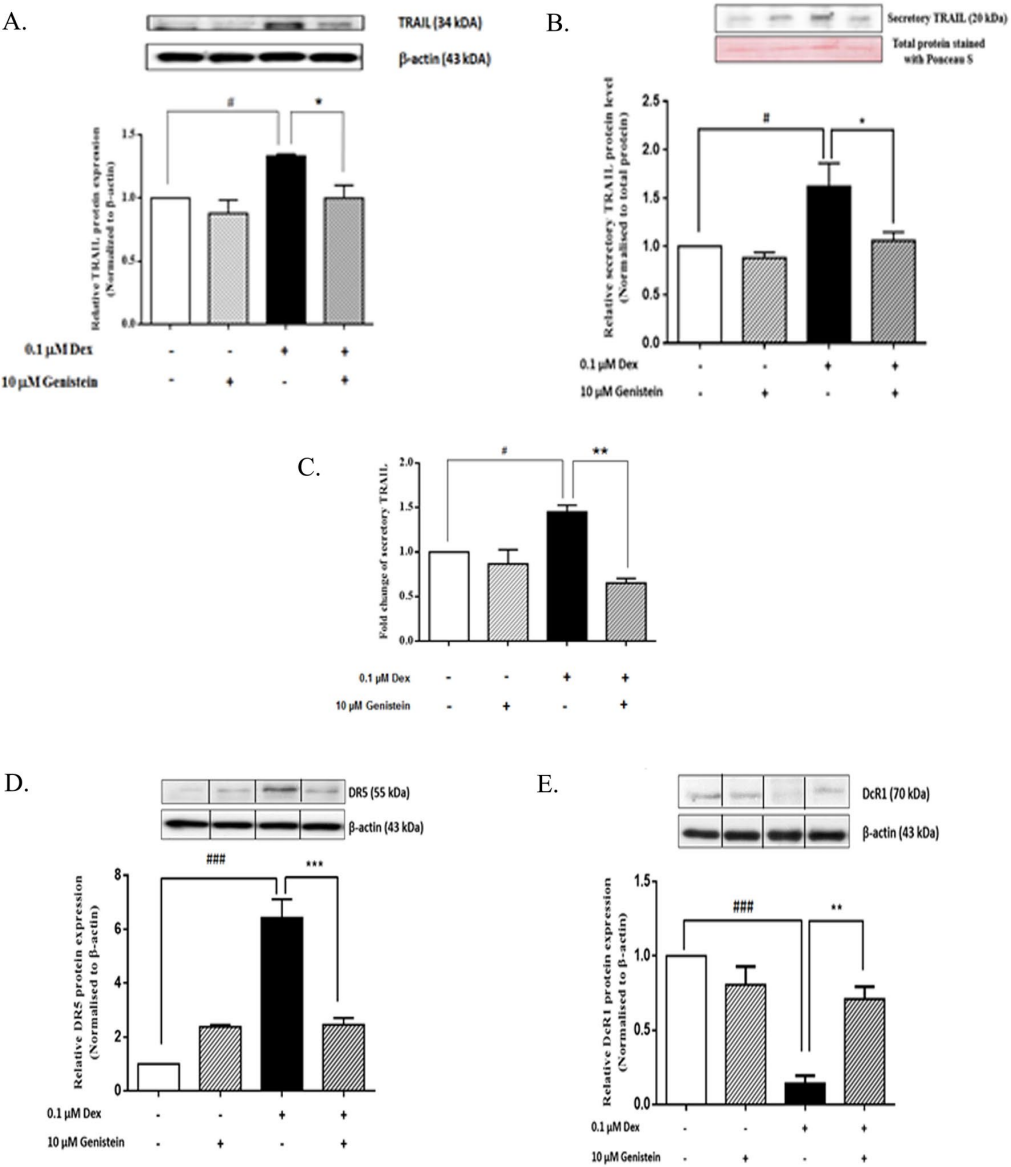
Effect of genistein on dexamethasone-induced TRAIL expression in isolated mouse islet and secretory TRAIL and TRAIL receptors from INS-1 cell line. (**A**) A representative Western blot analysis of TRAIL normalized to β-actin protein in mouse isolated islets. All bands were cropped and full-length blots are shown in supplementary Fig. 2. (**B**) Western blot analysis of soluble TRAIL protein normalized to total protein-stained with ponceau S dye in cultured media at 48 h. All bands were cropped and full-length blots are shown in supplementary Fig. 3. (**C**) ELISA analysis of soluble TRAIL protein. Raw data are shown in supplementary Fig. 4. (**D**) A representative Western blot analysis of DR5 normalized to β-actin protein at 48 h. All bands were cropped and full-length blots are shown in supplementary Fig. 5. (**E**) A representative Western blot analysis of DcR1 normalized to β-actin protein at 48 h. All bands were cropped and full-length blots are shown in supplementary Fig. 6. Data are expressed as mean ± SEM of 3–4 independent experiments. ###, *P* < 0.001, versus control; #, *P* < 0.05 versus control; ***, *P* < 0.001 versus 0.1 µM dexamethasone; **, P < 0.001 versus 0.1 µM dexamethasone; *, *P* < 0.05 versus 0.1 µM dexamethasone.

Upregulation of the TRAIL protein in isolated mouse islets was also observed when they were cultured with 0.1 µM dexamethasone. However, TRAIL protein expression in isolated mouse islets was significantly decreased when they were cultured in both 0.1 µM dexamethasone and 10 μM genistein when compared to the TRAIL protein expression observed in islets cultured in 0.1 µM dexamethasone alone (Fig. 2A).

### Genistein reduced dexamethasone-induced TRAIL protein secretion in INS-1 cells

The effect of genistein on dexamethasone-induced TRAIL protein secretion was also examined (Fig. 2B). Dexamethasone significantly increased TRAIL protein secretion compared to that of the control, and genistein significantly decreased dexamethasone-induced TRAIL protein secretion compared to that observed in the dexamethasone treatment alone. Genistein treatment alone had no effect on TRAIL protein secretion compared to that of the control.

Secretion of TRAIL protein after treatment of INS-1 cells with dexamethasone was confirmed by ELISA. Dexamethasone at 0.1 µM significantly increased TRAIL secretion of INS-1 cells as compared to that of the control condition. Genistein at 10 μM alone did not change the level of TRAIL secretion compared to that of the control condition. Combined treatment of genistein and dexamethasone could reduce TRAIL secretion to the same level as that of the control condition (Fig. 2C).

### Genistein decreased DR5 and restored DcR1 expression in dexamethasone-treated INS-1 cells

The effect of dexamethasone, genistein, or their combination on DR5 and DcR1 protein expression in INS-1 cells was examined by Western blot analysis. Dexamethasone significantly increased DR5 protein expression, but decreased DcR1 protein expression—both compared to control. The combined treatment of dexamethasone and genistein significantly decreased DR5 protein expression, but increased DcR1 protein expression compared to the expressions observed INS-1 cells cultured with dexamethasone alone. Genistein treatment alone had no significant effect on DR5 and DcR1 protein expression compared to control (Fig. 2D,E).

### Genistein reduced DR5 and restored DcR1 expression in dexamethasone-treated isolated mouse islets

**Figure 3:**
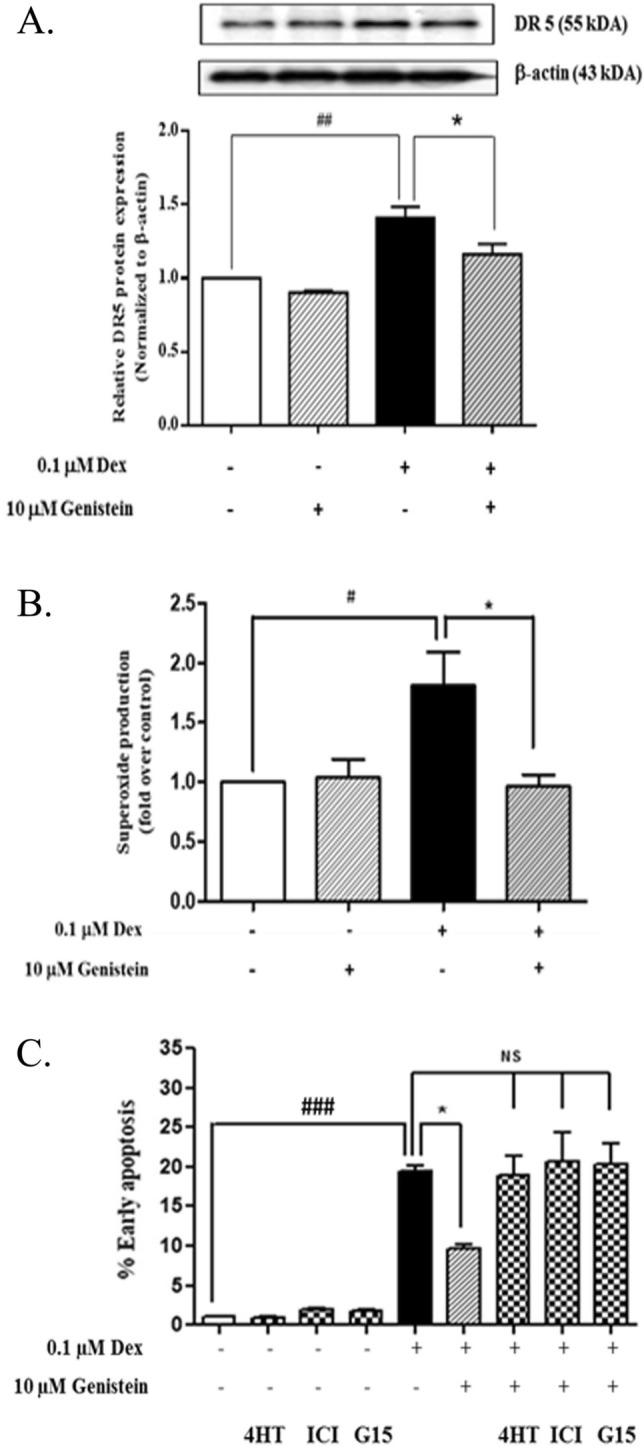
Effect of genistein on dexamethasone on DR5 expression in mouse isolated islets and on oxidative stress in INS-1 cell line. INS-1 cells and isolated mouse islets were treated with DMSO control or 0.1 μM dexamethasone with or without 10 μM genistein for 48 h. (**A**) A representative Western blot analysis of DR5 normalized to β-actin protein in mouse isolated islets. All bands were cropped and full-length blots are shown in supplementary Fig. 7. (**B**) Cellular superoxide production was determined by NBT assay. (**C**) A representative of AnnexinV/PI dot plot of the effect of dexamethasone and with or without 10 μM genistein in the presence or absence of estrogen receptors inhibitors including, estrogen α receptor inhibitor (4HT), estrogen α and β receptor inhibitor (ICI) or estrogen membrane receptor inhibitor (G15) for 72 h. Data are expressed as mean ± SEM of 3–4 independent experiments. ###, *P* < 0.001 versus control, ##, *P* < 0.001 versus control, #, *P* < 0.05 versus control; *, *P* < 0.05 versus 0.1 µM dexamethasone.

The effect of dexamethasone, genistein, or their combination on DR5 protein expression in isolated mouse islets was also examined (Fig. 3A). Similar to what was observed in INS-1 cell line, dexamethasone significantly increased DR5 protein expression in isolated mouse islets compared to control. The combined treatment of dexamethasone and genistein significantly decreased DR5 protein expression in isolated mouse islets compared to the expression observed in dexamethasone treatment alone. Genistein treatment alone had no effect on DR5 protein expression in isolated mouse islets when compared to that of the control.

### Genistein decreased dexamethasone-induced cellular superoxide production

To investigate the antioxidant effect of genistein on dexamethasone-induced oxidative stress in INS-1 cells, cellular superoxide level was determined by NBT assay (Fig. 3B). Dexamethasone significantly increased superoxide level compared to control. Combination dexamethasone and genistein treatment significantly reduced superoxide level compared to that observed in dexamethasone treatment alone. Genistein treatment alone had no effect on superoxide level compared to control.

### Genistein reduced dexamethasone-induced INS-1 apoptosis via nuclear and membrane estrogen receptors

To examine whether genistein could prevent pancreatic β-cell apoptosis via estrogen receptors (ER), INS-1 cells were cultured in 0.1 µM dexamethasone in the presence or absence of ERα inhibitor—4HT, ERα and ERβ inhibitor—ICI 182,780, and ER membrane receptor inhibitor—G15, before measuring the apoptotic cells by Annexin V FITC/PI staining. Dexamethasone significantly increased cell apoptosis, whereas combined treatment of dexamethasone and genistein significantly decreased cell apoptosis. All of ER receptor inhibitors abolished the protective effect of genistein. Combined treatments of dexamethasone, genistein and each of ER receptor inhibitors brought cell apoptosis back to similar level as that of the treatment with dexamethasone. The treatment with either nuclear or membrane ER inhibitor alone had no effect on cell apoptosis compared to that of the control condition (Fig. 3C).

### Effect of genistein on dexamethasone-induced extrinsic apoptotic pathway in INS-1 cells

**Figure 4:**
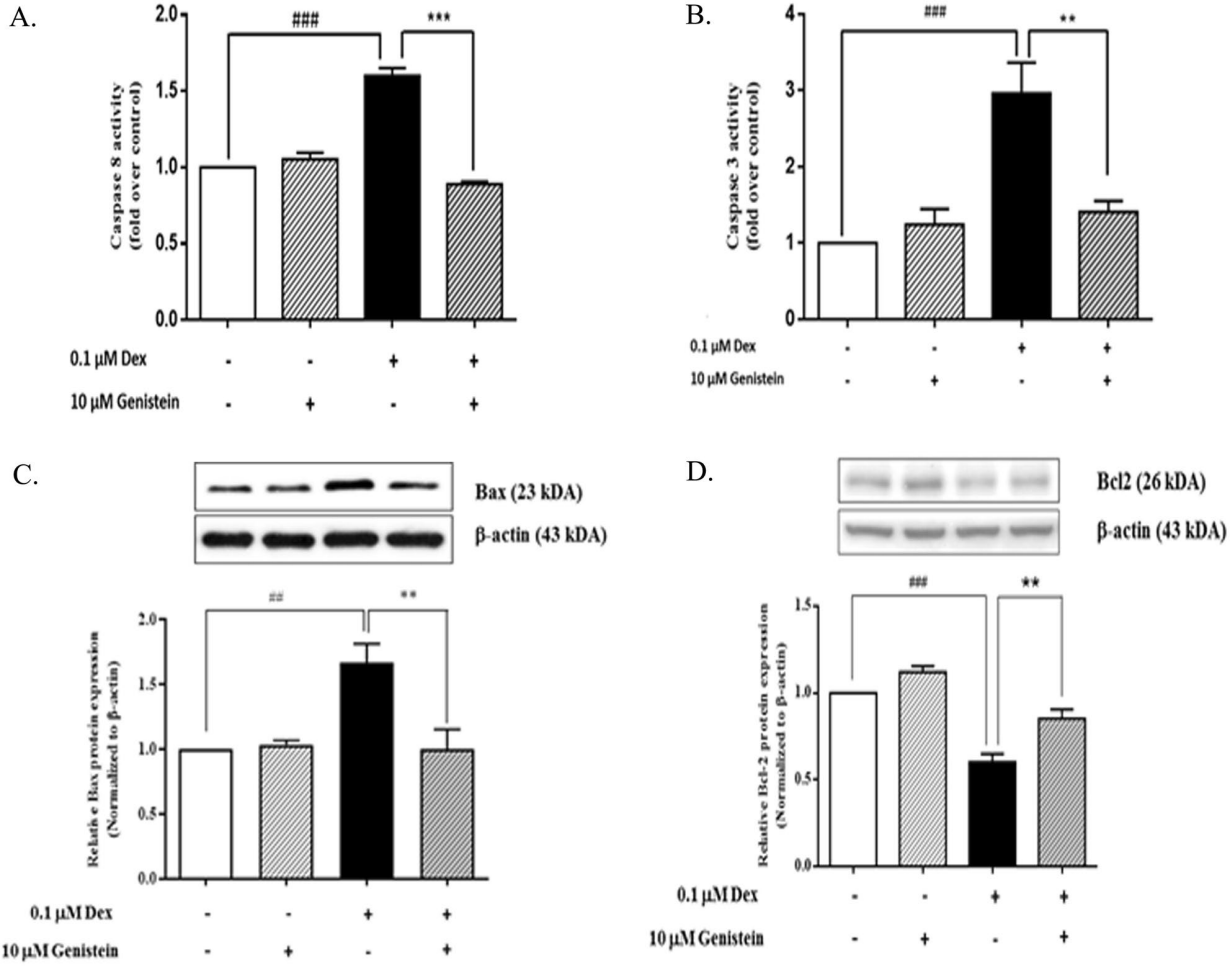
Effect of genistein on dexamethasone-induced dexamethasone on cleaved caspase-3, caspase-8 activity, Bax and Bcl2 in INS-1 cell line. INS-1 cells were treated with 0.1 μM dexamethasone with or without 10 μM genistein. (**A**) Relative level of caspase-8 activity at 48 h. (**B**) Relative level of caspase-3 activity at 48 h. (**C**) A representative Western blot analysis of the pro-apoptotic protein, Bax, normalized to β-actin protein in INS-1 cells at 48 h. All bands were cropped and full-length blots are shown in supplementary Fig. 8. (**D**) A representative Western blot analysis of the anti-apoptotic protein, Bcl-2, normalized to β-actin protein in INS-1 cells at 48 h. All bands were cropped and full-length blots are shown in supplementary Fig. 9. Data are expressed as mean ± SEM of 3–4 independent experiments. ###, *P* < 0.001 versus control; ##, *P* < 0.01 versus control; ***, *P* < 0.001 versus 0.1 µM dexamethasone; **, P < 0.001 versus 0.1 µM dexamethasone.

To evaluate the effect of genistein on the dexamethasone-induced extrinsic apoptotic pathway in INS-1 cells, caspase-8 and caspase-3 activities were examined by proteolytic luminescence assay (Fig. 4A,B). Caspase-8 and caspase-3 activities were both significantly increased in INS-1 cells treated with dexamethasone. Combination dexamethasone and genistein treatment significantly reduced caspase-8 and caspase-3 activities compared to those observed in dexamethasone treatment alone.

### Effect of genistein on dexamethasone-induced intrinsic apoptotic pathway in INS-1 cells

To analyze the effect of genistein on the dexamethasone-induced intrinsic apoptotic pathway in INS-1 cells, the pro-apoptotic Bax protein and the anti-apoptotic Bcl protein were examined by Western blot analysis (Fig. 4C,D). Dexamethasone treatment alone increased Bax protein expression compared to control. Combined treatment of dexamethasone and genistein reduced Bax protein expression compared to that observed in dexamethasone treatment alone (Fig. 4C). In contrast, dexamethasone treatment alone significantly reduced Bcl-2 protein expression compared to control (Fig. 4D). Combination dexamethasone and genistein treatment significantly increased Bcl-2 protein expression compared to that observed in dexamethasone treatment alone. Genistein treatment alone had no significant effect on Bax or Bcl-2 protein expression.

### Genistein reduced dexamethasone-induced NF-κB p65 phosphorylation in INS-1 cells

**Figure 5:**
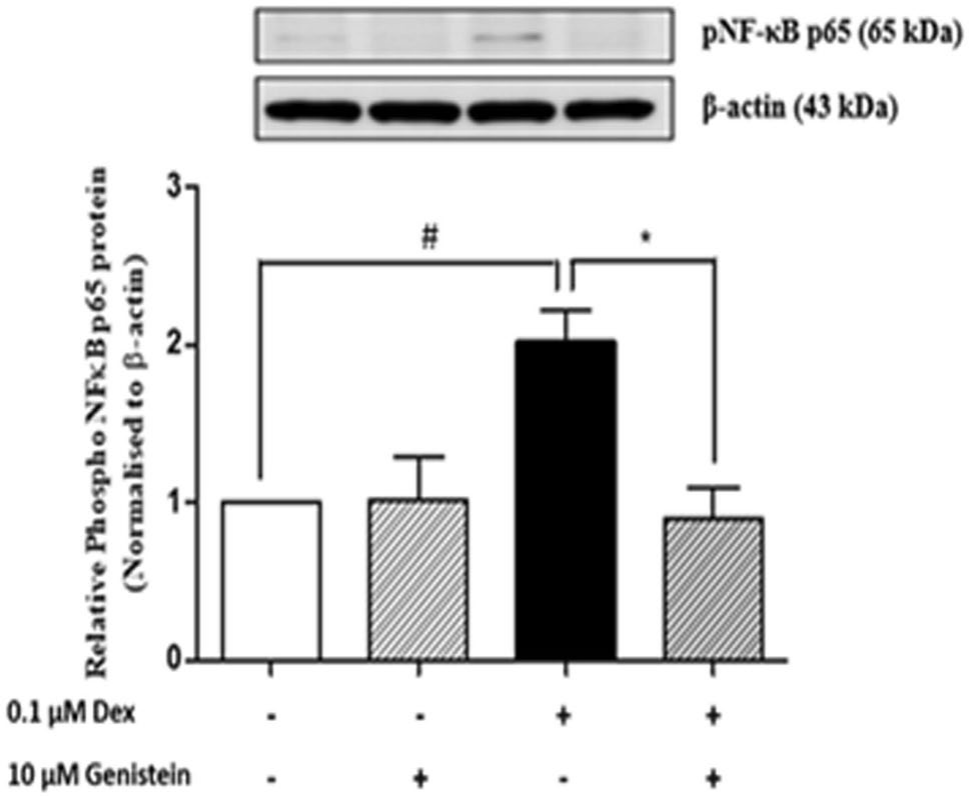
Effect of genistein on dexamethasone-induced NF-κB activation. INS-1 cells were treated with 0.1 μM dexamethasone with or without 10 μM genistein of 48 h. A representative Western blot analysis of phosphorylated NF-κB p65 protein normalized to β-actin protein in INS-1 cells. All bands were cropped and full-length blots are shown in supplementary Fig. 10. Data are expressed as mean ± SEM of 3 independent experiments. #, *P* < 0.05 versus control; *, *P* < 0.05 versus 0.1 μM dexamethasone.

To investigate the effect of genistein on dexamethasone-induced NF-κB p65 phosphorylation, phosphorylated NF-κB p65 protein was examined by Western blot analysis. Dexamethasone treatment alone significantly increased NF-κB p65 phosphorylation (Fig. 5), whereas combination treatment with dexamethasone and genistein significantly decreased NF-κB p65 phosphorylation. Genistein treatment alone had no effect on NF-κB p65 phosphorylation compared to control.

## Discussion

The protective effect of genistein on dexamethasone-induced pancreatic β-cell apoptosis is unknown, and it has previously been demonstrated by our group that dexamethasone-induced pancreatic β-cell apoptosis is associated with upregulated expression of TRAIL and DR5. We, therefore, hypothesized that genistein protects against dexamethasone-induced pancreatic β-cell apoptosis by downregulating TRAIL and DR5 protein expression leading to inhibition of this apoptotic pathway. To test this hypothesis, we investigated the effects of genistein on dexamethasone-induced pancreatic β-cell apoptosis in cultured INS-1 cell line and isolated mouse islets. The results of the present study confirmed and extended our previous findings that dexamethasone-induced pancreatic β-cell apoptosis is associated with upregulation of TRAIL, DR5, and superoxide production, but with downregulation of DcR1. Dexamethasone also upregulated the expression of extrinsic and intrinsic apoptotic proteins, including Bax, NF-κB, caspase 8, and caspase 3, but downregulated the expression of the anti-apoptotic Bcl-2 protein. However, dexamethasone-induced pancreatic β-cell apoptosis was protected by genistein. Treatment of INS-1 cells with genistein reduced the effects of dexamethasone on the expressions of TRAIL, DR5, DcR1, superoxide production, Bax, Bcl-2, NF-κB, caspase 8 and caspase 3. Combination treatment with dexamethasone and genistein also reduced the expression of TRAIL and DR5 in isolated mouse islets.

Our results also showed that dexamethasone increased reactive oxygen species (ROS). This finding correponds with the results of previous studies that showed that dexamethasone generated cellular ROS in macrophages, osteoblasts, and testicular germ cells^25–27^. The increased intracellular ROS level could activate NF-κB^28^. Dexamethasone was shown to activate NF-κB and apoptosis in muscle cells^29^. This effect was also observed in pancreatic β-cells, which was associated with pancreatic β-cell apoptosis^12,13^. Interestingly, it was reported that NF-κB binding sites are present within the promoters of both *TRAIL* and *DR5* genes^30,31^, and that NF-κB activation promoted *TRAIL* and *DR5* expression^32^. Thus, it is possible that dexamethasone upregulates TRAIL and DR5 expression via the activation of NF-κB. Previous study found that TRAIL and DR5 could stimulate NF-κB^33^. We also demonstrated that the addition of genistein to cultured cells treated with dexamethasone significantly decreased the superoxide level when compared to the level observed in cells treated with dexamethasone alone. Genistein is known to have higher antioxidant activity than other soy phytoestrogens^34^. Genistein was shown to increase the activities of antioxidant enzymes, including superoxide dismutase, catalase, glutathione peroxidase, and glutathione reductase^35,36^. Our results also showed that genistein inhibited NF-κB p65 phosphorylation. This corresponds with the results of previous studies that found that genistein inhibited NF-κB-upregulated inducible nitric oxide synthase (iNOS) expression in pancreatic β-cells^22^. Genistein deactivated NF-κB in low-density lipoprotein receptor (LDLR) knockout mice^37^, and inhibited NF-κB p65 activation to protect against lipopolysaccharide (LPS)-induced lung injury in rat^38^. This study further investigated whether genistein exerted it effects via ER receptors or not. Our results showed that both nuclear and membrane ER inhibitors diminished the protective effect of genistein against dexamethasone. Furthermore, our previous study demonstrated that prunetein, a flavanoid that has dominant tyrosine kinase inhibitor, also have similar effect as genistein^39^. Thus, genistein protected against dexamethasone-induced β-cell apoptosis via its antioxidant, estrogenic effects, and inhibitory effect on tyrosine kinase.

**Figure 6:**
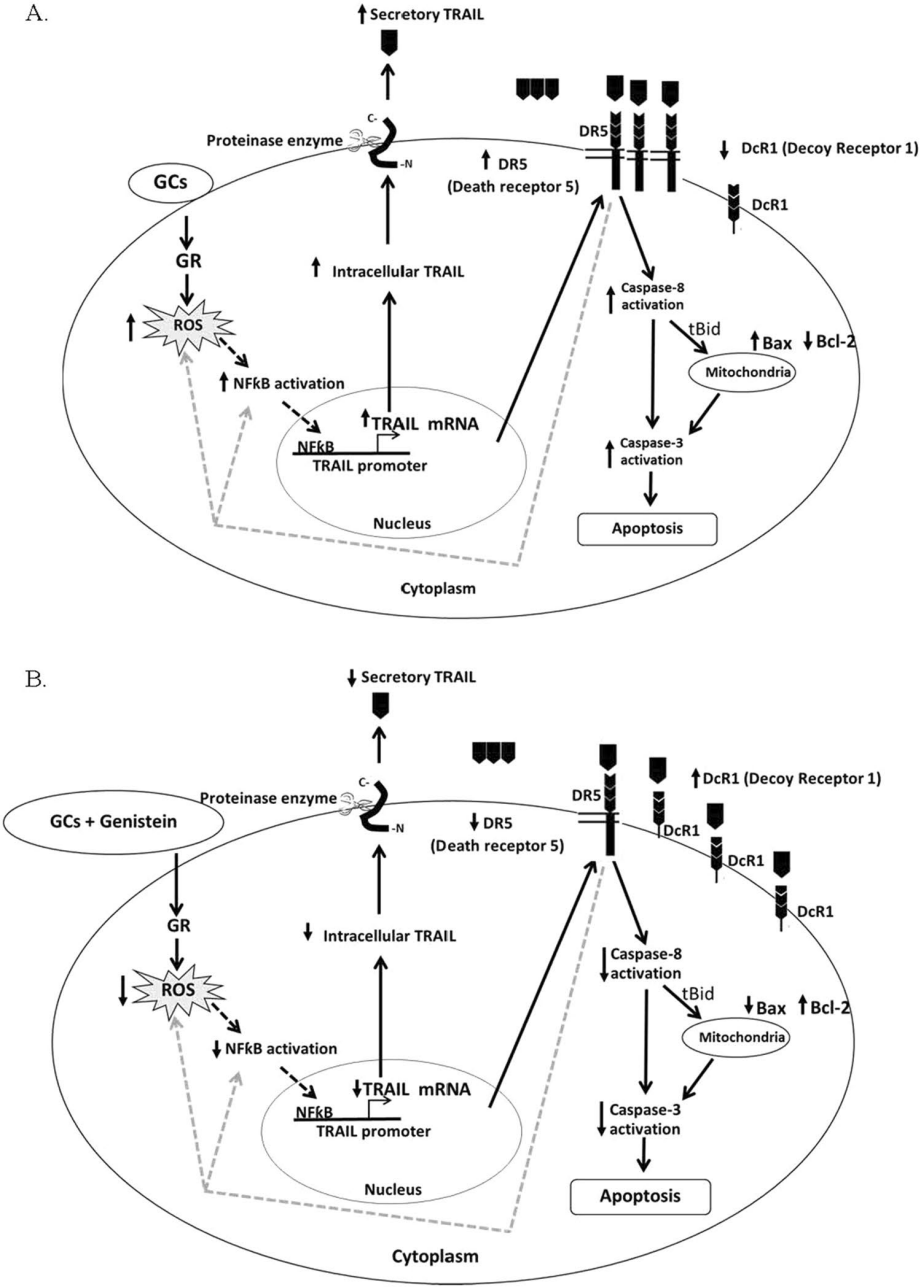
Possible mechanism of genistein protects against GC-induced pancreatic β-cell apoptosis through TRAIL pathway. (**A**) Possible mechanism of GCs induces pancreatic β-cell apoptosis through TRAIL pathway. (**B**) Possible mechanism of genistein exerts to protect pancreatic β-cell apoptosis from dexamethasone by inhibiting TRAIL pathway.

Another finding from this study was that dexamethasone increased secretory TRAIL. Binding between secretory TRAIL and DR5 induced cell apoptosis via activation of caspase-8. Activated caspase-8 then induced cell apoptosis via the extrinsic apoptotic pathway involved in direct activation of caspase-3; however, induction of the intrinsic mitochondrial apoptotic pathway was also observed^40,41^. Our results demonstrated that combination dexamethasone and genistein treatment reduced cell apoptosis and decreased the expression of the caspase-8, caspase-3, and Bax proteins. This finding is consistent with previous studies that showed that genistein could protect against apoptosis in different cell types^42–44^. It was also reported that genistein protected against cytokine-mediated toxicity-induced apoptosis of the rat insulinoma cell line RINm5F^22^. Cytokines could also be used as markers for other complications of steroid^45^. Moreover, genistein protected against neuronal cell death due to aggregated amyloid β damage^46^. Genistein reduced caspase-3 and caspase-8 activities in other cell types, including primary rat granulosa cells^47^ and testicular cells^48^. Genistein also reduced Bax, but induced Bcl2 expression in neuronal cells and gastric mucosal cells^42,49^. Thus, it is possible that genistein protected against dexamethasone-induced pancreatic β-cell apoptosis via inhibition of both extrinsic and intrinsic apoptotic pathways (Fig. 6A,B).

The results of this study demonstrate that genistein protects against pancreatic β-cell glucocorticoid-induced apoptosis via the inhibition of TRAIL and DR5 expression. Molecular mechanisms that link the effects of genistein to TRAIL expression warrant further investigation.

## Materials and methods

### Animal model

Male outbred 8–12-week-old ICR mice were purchased from the National Laboratory Animal Center, Mahidol University, Bangkok, Thailand. The animal experimentation protocol was approved by the Institutional Animal Care and Use Committee of the Faculty of Medicine Siriraj Hospital, Mahidol University (approval no. SI-ACUP 001/2559), which was approved on 29th February 2016. All the experiment followed the arrive guidelines. Eight-week-old male ICR mice were acclimatized for 1 week and then randomly assigned to either a treatment group or a control group, and there were 5–6 mice in each group. The mice in all groups were maintained in a 12-h light/dark cycle environment at 25 ± 2 °C and 60% humidity. Mice were housed 5–6 animals per cage with wood chip bedding, and chow pellet was available ad libitum (Perfect Companion Group Co., Ltd., Bangkok, Thailand).

### Mouse pancreatic islet isolation and culture

Pancreatic islets were isolated by collagenase digestion using a method modified from that of Lacy & Kostianovsky (Lacy and Kostianovsky, 1967) and Gotoh (Gotoh et al., 1985). Briefly, mouse were euthanized with CO_2_ for 2 min then pancreas was infused with collagenase-P and digested at 37 °C. Islets were separated using histopaque gradient and hand-picked under a stereomicroscope. Isolated islets were cultured in Roswell Park Memorial Institute (RPMI) 1640 Medium supplemented with heated-activated fetal bovine serum (FBS), penicillin, and streptomycin at 37 °C in 5% CO2. The culture medium was changed every 2 days. A total of 300–500 islets were cultured with or without 0.1 µM dexamethasone in the presence or absence of 10 µM genistein for 7 days.

### INS-1 cell culture

Rat insulinoma (INS-1) cell line was cultured in RPMI 1640 Medium supplemented with 10% FBS, 100 U/ml penicillin, and 100 µg/ml streptomycin at 37 °C in humidified air containing 5% CO2. The culture medium was changed every 2 days. The cells were cultured in medium without or with 0.1 µM dexamethasone, and in the absence or presence of 10 μM genistein. All the performed experiment done in the study with the followed guidelines and regulations.

### Measurement of intracellular superoxide generation

Superoxide production was detected by nitroblue tetrazolium (NBT) assay. INS-1 cells were treated with 0.1 µM dexamethasone in the absence or presence of 10 μM genistein for 48 h. After incubation, the cells were incubated with NBT for 90 min. The cells were then lysed in potassium hydroxide (KOH). The released insoluble formazan was dissolved in dimethyl sulfoxide (DMSO). Superoxide production was measured as optical density (OD) at a wavelength of 630 nm using a PowerWave Microplate Scanning Spectrophotometer (BioTek Instruments, Inc., Winooski, VT, USA).

### Analysis of cell apoptosis by annexin V-FITC/PI staining

INS-1 cells were treated with 0.1 µM dexamethasone in the absence or presence of 10 μM genistein. After incubation for 72 h, the cells were collected and stained with propidium iodine (PI), and cell apoptosis was detected using an FITC Annexin V Apoptosis Detection Kit (BD Biosciences, San Jose, CA, USA). The apoptotic cells were then analyzed using a FACSort Flow Cytometer (Becton, Dickinson and Company, San Jose, CA, USA).

### Caspase-3 activity assay

Caspase-3 assay was performed according to the manufacturer’s protocol (Promega Corporation, Madison, WI, USA). INS-1 cells were seeded into a 96-well plate and allowed to attach overnight. The INS-1 cells were cultured with 0.1 µM dexamethasone in the absence or presence of 10 μM genistein. At 72 h after treatment, 100 μl of caspase-Glo reagent was added to each well and gently mixed on a plate shaker for 30 s. The plates were then incubated at 37 °C for 30 min in the dark. A substrate for luciferase (aminoluciferin) was released after caspase-3 enzyme cleavage. Luminescence was measured by plate-reading luminometer (Synergy H1 Hybrid Multi-Mode Microplate Reader; Bio-Tek Instruments). The change in luminescence signal is directly proportional to caspase-3 activity.

### Caspase-8 activity assay

Caspase-8 assay was performed according to the manufacturer’s protocol (Promega). INS-1 cells were seeded into a 96-well plate and allowed to attach overnight. The INS-1 cells were cultured with 0.1 µM dexamethasone in the absence or presence of 10 μM genistein. At 48 h after treatment, 100 μl of caspase-Glo reagent was added to each well and gently mixed on a plate shaker for 30 s. The plates were then incubated at 37 °C for 30 min in the dark. A substrate for luciferase (aminoluciferin) was released after caspase-8 enzyme cleavage. Luminescence was measured by a plate-reading luminometer (Synergy H1 Hybrid Multi-Mode Microplate Reader; Bio-Tek Instruments). The change in luminescence signal is directly proportional to caspase-8 activity.

### RNA isolation and real-time quantitative reverse transcription-polymerase chain reaction (real-time RT-qPCR)

INS-1 cells were cultured with 0.1 µM dexamethasone in the absence or presence of 10 μM genistein for 48 h. The total RNA of INS-1 cells was extracted using a High Pure RNA Isolation Kit (Roche Diagnostics, Basel, Switzerland). RNA concentration was measured using a NanoDrop (ND)-1000 Spectrophotometer (NanoDrop Technologies LLC, Wilmington, DE, USA). Reverse transcription was performed using 1 μg of RNA and a reagent from a SuperScript III Reverse Transcription Kit with random hexamer primer (Invitrogen Corporation, Carlsbad, CA, USA). Real-time RT-qPCR was then performed using SYBR Green.

Reaction Mix and a Roche LightCycler 480 Instrument (Roche Diagnostics). A pair of specific primers for rat TRAIL included forward primer: 5′ TGATGAAGAGTGCCAGAAAATAGC 3′ and reverse primer: 5′ CCAGGTCCATCAAATGCTCA 3′. The primers used for rat β-actin were forward primer: 5′ ATGAAGTGACGTTGACA 3′ and reverse primer: 5′ CCTGAAGCATTTGCGGTGCACGATG 3′. The threshold cycles (Ct) of TRAIL and β-actin genes were measured, and the difference between their ∆Ct was calculated. Relative expression was then calculated using the 2-∆∆Ct method, and the results was compared to that of the control group.

### Western blot analysis

INS-1 cells and mouse pancreases were lysed in radioimmunoprecipitation assay (RIPA) buffer (Pierce Biotechnology Corporation, Rockford, IL, USA), and quantified for total protein using a Micro BCA Protein Assay Kit (Pierce Technology). Total proteins were separated by sodium dodecyl sulfate polyacrylamide gel electrophoresis (SDS-PAGE) and then transferred to a polyvinylidene fluoride membrane (Bio-Rad Laboratories, Hercules, CA, USA). The membrane was then blocked with 5% skimmed milk. The membrane was incubated overnight at 4 °C with one of the following primary antibodies: mouse monoclonal anti-TRAIL, goat polyclonal anti-DR5, goat polyclonal anti-DcR1, mouse monoclonal anti-Bax, rabbit polyclonal anti-Bcl-2, mouse monoclonal anti-NF-κB, goat polyclonal anti-α-tubulin, mouse monoclonal anti-β-actin (all from Santa Cruz Biotechnology, Dallas, TX, USA), or mouse monoclonal anti-GAPDH (Cell Signaling Technology, Danvers, MA, USA). The membranes were then washed and incubated with horseradish peroxidase (HRP)-conjugated secondary antibody (Santa Cruz Biotechnology). Protein bands were detected by enhanced chemiluminescence (Pierce Biotechnology). The band intensities of the proteins were analyzed using ImageJ densitometry software (version 1.43; National Institute of Health, Bethesda, MD, USA).

### Secretory protein collection

Soluble TRAIL in culture media was collected by using a Vivaspin spin column containing an ultrafiltration membrane (Cytiva, Marlborough, MA, USA). INS-1 cells were treated with 0.1 μM dexamethasone in the absence or presence of 10 μM genistein. At 48 h after the treatment, the culture medium was transferred to a membrane ultrafiltration tube with a size of 6 ml (5 kDa molecular weight cutoff [MWCO]) and centrifuged for 20 min. After centrifugation, the solution in the filtrate container was discarded. The concentrated protein sample in the concentrate pocket was collected with a pipette. The sample’s concentration was measured by using a Micro BCA Protein Assay Kit (Pierce Biotechnology).

### Enzyme linked immunosorbent assay

Enzyme linked immunosorbent assay (ELISA) kit for TRAIL protein was purchased from CLOUD-CLONE CORP. (CCC, USA). INS-1 cells were treated with 0.1 µM dexamethasone in the presence or absence of 10 μM genistein. After incubation for 48 h, TRAIL protein in the supernatants was detected by ELISA according to the protocol provided by the manufacturer (CCC, USA). Optical absorption at 450 nm was measured, and the acquired data were analyzed.

### Statistical analysis

Data are expressed as mean ± standard error of mean (SEM), and a p-value less than 0.05 was considered to be statistically significant. Differences between datasets were determined by one-way analysis of variance (ANOVA) followed by Tukey’s post hoc test.

## Supplementary Information

Supplementary Information.

## Data Availability

All data generated or analyzed during this study are included in this published article and its supplementary information files.

## Abbreviations

Bax: BCL-2-associated X protein
BCA: Bicinchoninic acid
Bcl-2: B-cell lymphoma 2
DcR1: Decoy receptor 1
DcR2: Decoy receptor 2
DR4: Death receptor 4
DR5: Death receptor 5
GC: Glucocorticoid
GR: Glucocorticoid receptor
HRP: Horseradish peroxidase
INS-1: Rat insulinoma cell line
NBT: Nitroblue tetrazolium
NFAT: Nuclear factor of activated T-cells
NF-κB: Nuclear factor kappa B
OD: Optical density
OPG: Osteoprotegerin
PI: Propidium iodide
ROS: Reactive oxygen species
STAT1: Signal transducer and activator of transcription
TRAIL: Tumor necrosis factor-related apoptosis-inducing ligand
