# Supplementary material for: Cytoprotective effect of genistein against dexamethasone-induced pancreatic β-cell apoptosis
Source: Sci Rep. 2022 Jul 28;12:12950. doi: 10.1038/s41598-022-17372-z (PMC9334585; doi:10.1038/s41598-022-17372-z)
Supplement: Supplementary file 1 — Supplementary Information. [file 41598_2022_17372_MOESM1_ESM.pdf]

Cytoprotective Effect of Genistein Against Dexamethasone-Induced Pancreatic β-Cell Apoptosis.

Kanchana Suksri[a], Namoiy Semprasert[a], Thawornchai Limjindaporn[b], Pa-thai Yenchitsomanus[c], Suwattanee Kooptiwut*[a]

[a]*Division of Endocrinology, Department of Physiology, Faculty of Medicine Siriraj Hospital, Mahidol University, Bangkok, Thailand*
[b]*Department of Anatomy, Faculty of Medicine Siriraj Hospital, Mahidol University, Bangkok, Thailand*
[c]*Division of Molecular Medicine, Research Department, Faculty of Medicine Siriraj Hospital, Mahidol University, Bangkok, Thailand*

Corresponding author:
Suwattanee Kooptiwut, MD, PhD
Associate Professor of Physiology
Division of Endocrinology, Department of Physiology
Faculty of Medicine Siriraj Hospital, Mahidol University
2 Wanglang Road, Bangkoknoi, Bangkok 10700, Thailand
Tel: (+66) 2-419-9720; Fax: (+66) 2-411-5009
E-mail: S_kooptiwut@hotmail.com

Original blot figure (Figure 1D) (INS-1)

TRAIL (34 kDa):

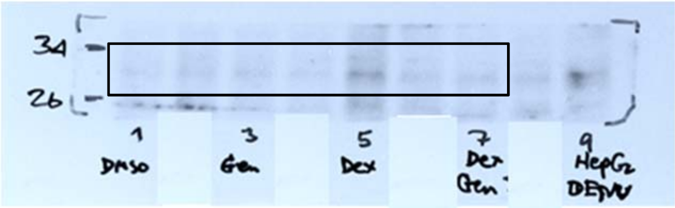

β-actin (43 kDa):

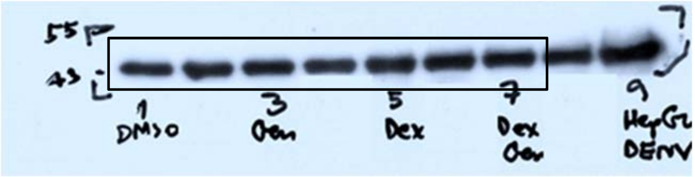

TRAIL (34 kDa):

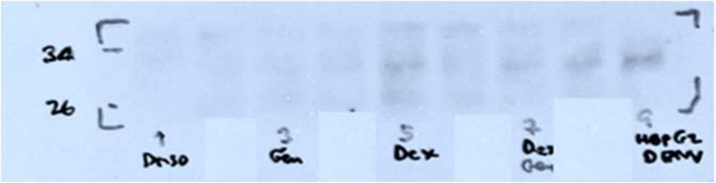

β-actin (43 kDa):

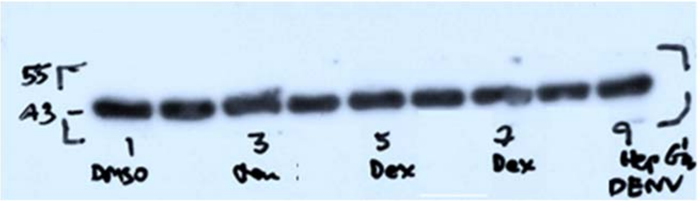

TRAIL (34 kDa):

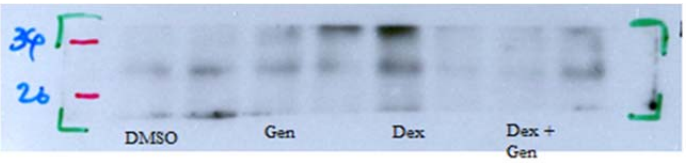

β-actin (43 kDa):

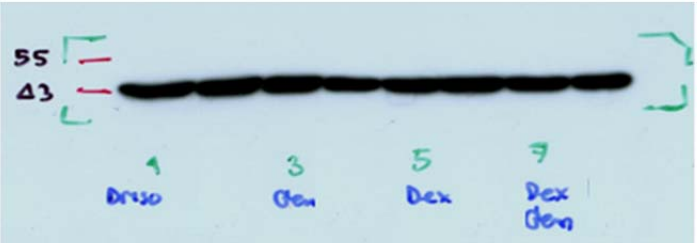

Supplement figure 1

Original blot figure (Figure 2A) (mouse islets)

TRAIL (34 kDa):

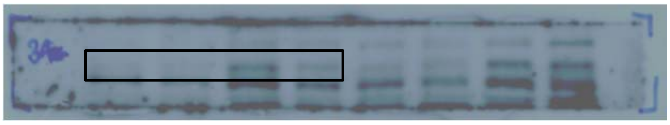

β-actin (43 kDa):

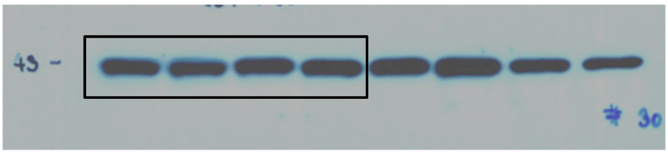

TRAIL (34 kDa):

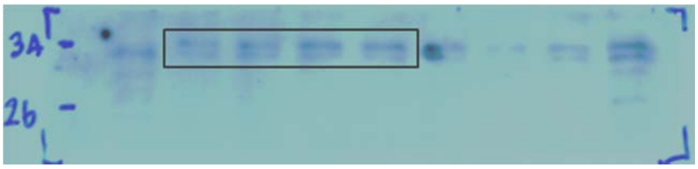

β-actin (43 kDa):

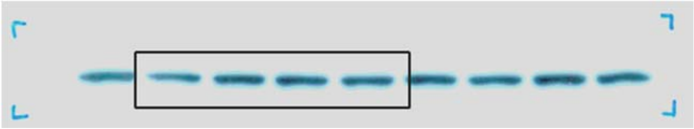

TRAIL (34 kDa):

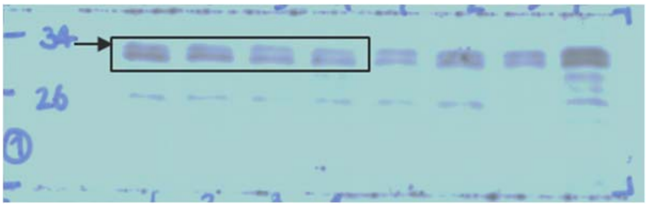

β-actin (43 kDa):

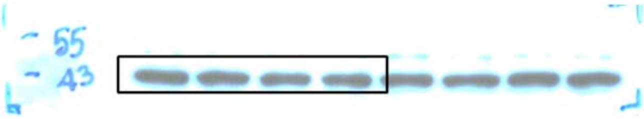

Original blot figure (Figure 2B)

Secretory TRAIL (20 kDa):

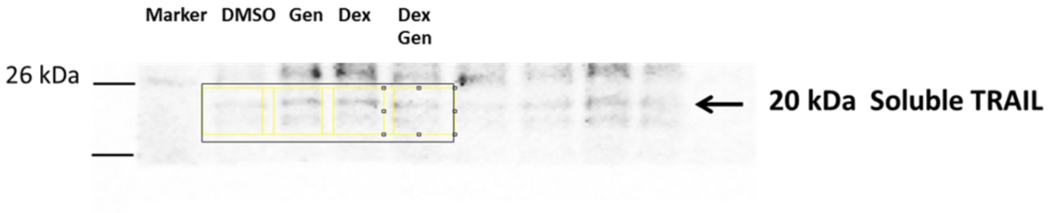

Total protein

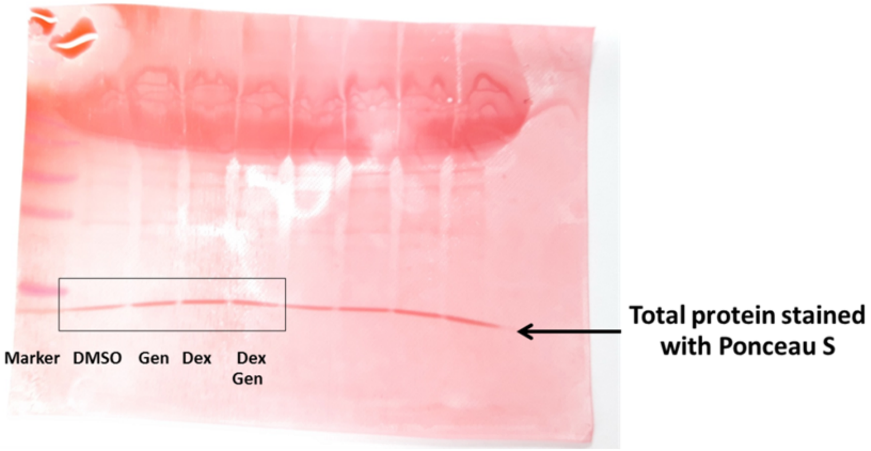

Secretory TRAIL (20 kDa):

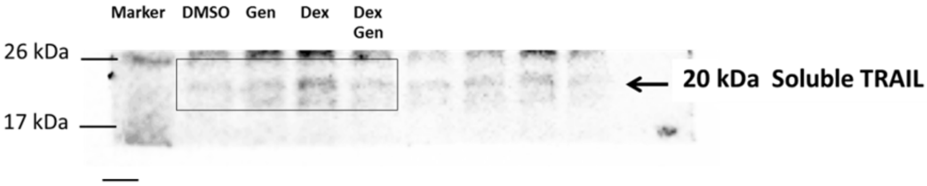

Total protein

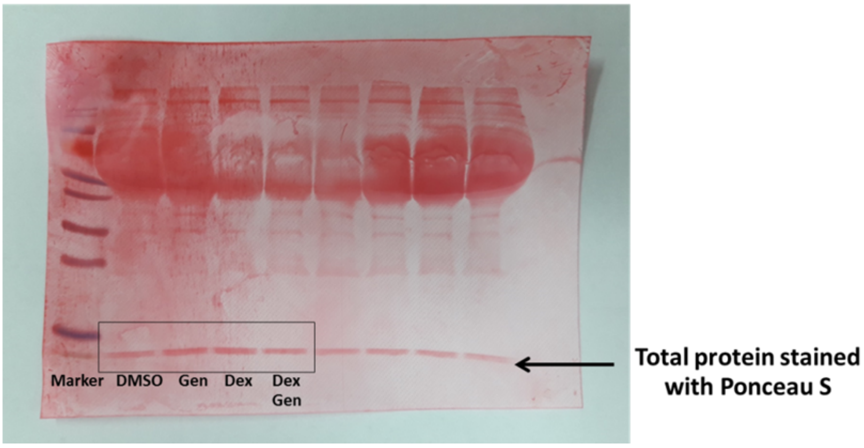

Supplement figure 3

ELISA data (Figure 2C)

Experiment 1

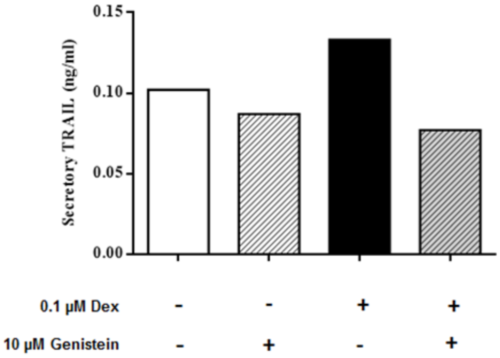

Experiment 2

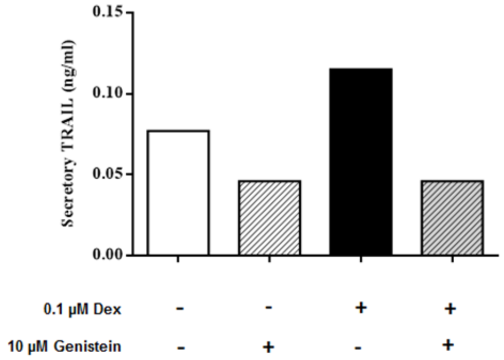

Experiment 3

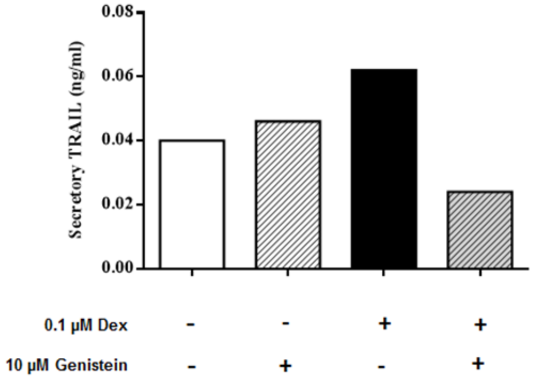

Result of 3 experiments

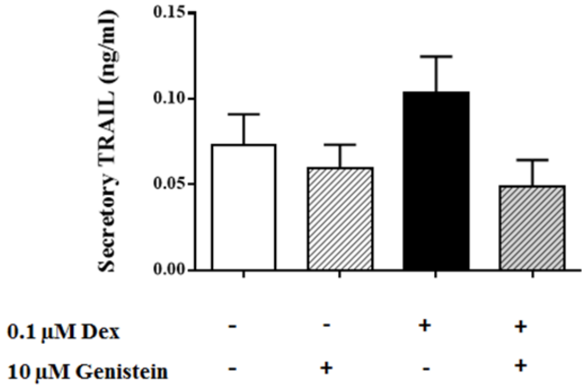

Supplement figure 4

Original blot figure (Figure 2D) (INS-1)

DR5 (55 kDa):

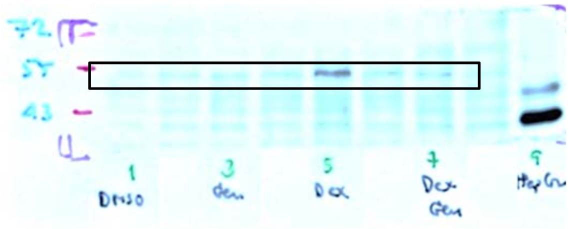

β-actin (43 kDa):

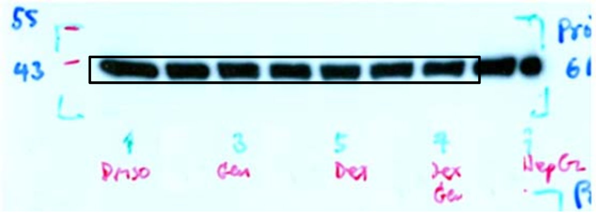

DR5 (55 kDa):

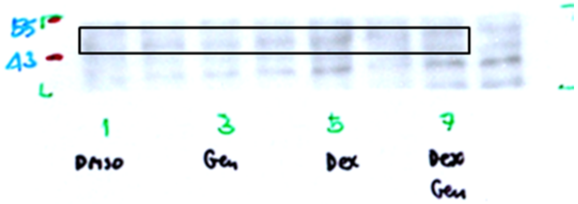

β-actin (43 kDa):

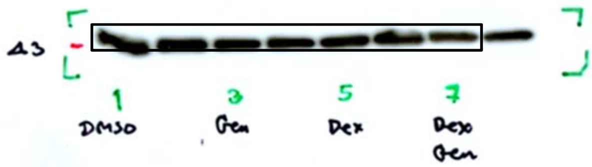

DR5 (55 kDa):

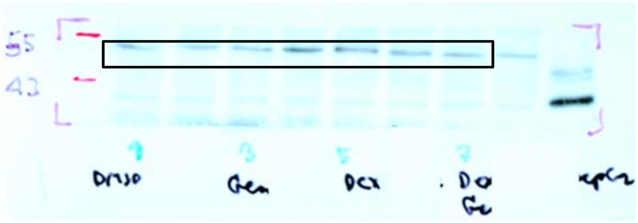

β-actin (43 kDa):

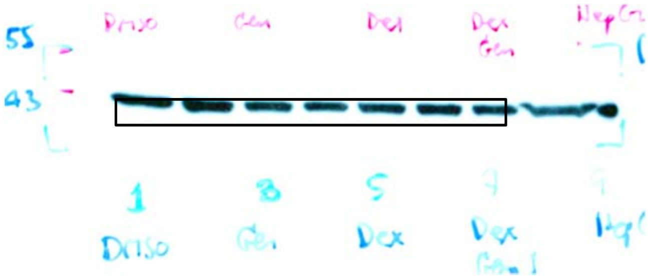

Supplement figure 5

Original blot figure (Figure 2E) (INS-1)

DcR1 (70 kDa):

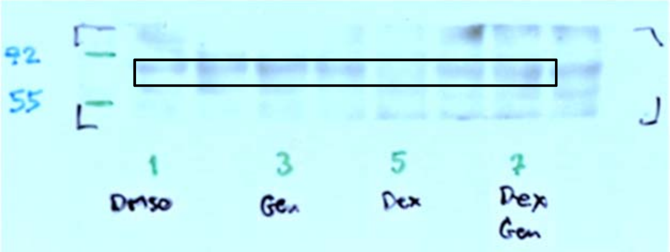

β-actin (43 kDa):

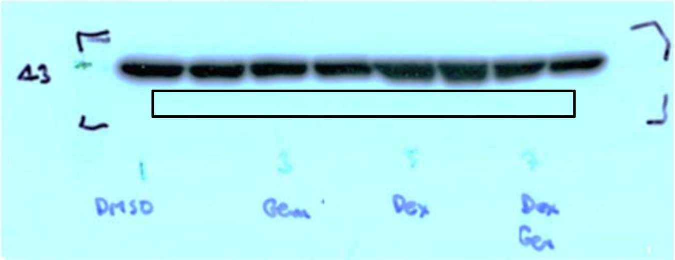

DcR1 (70 kDa):

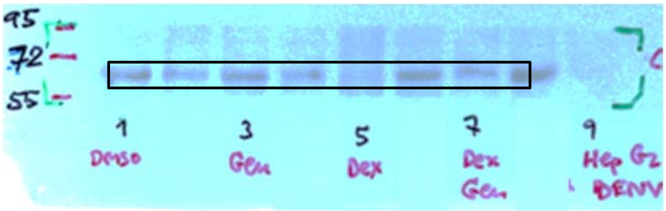

β-actin (43 kDa):

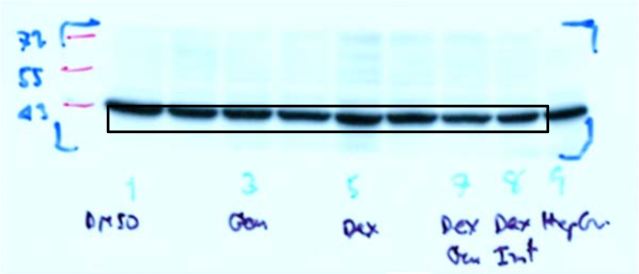

DcR1 (70 kDa):

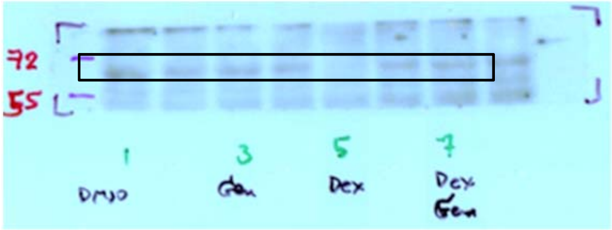

β-actin (43 kDa):

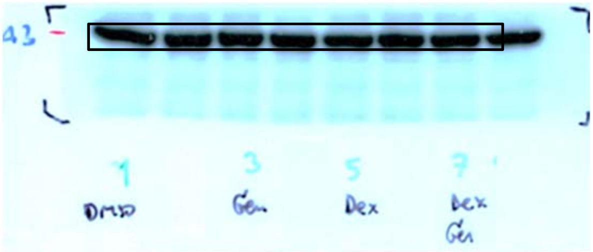

Supplement figure 6

Original blot figure (Figure 3A) (mouse islets)

DR5 (55 kDa):

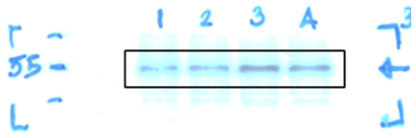

β-actin (43 kDa):

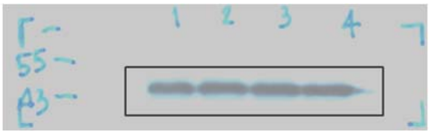

DR5 (55 kDa):

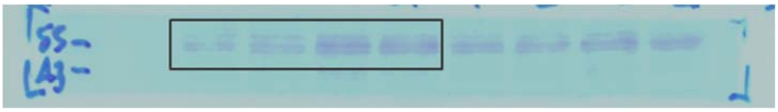

β-actin (43 kDa):

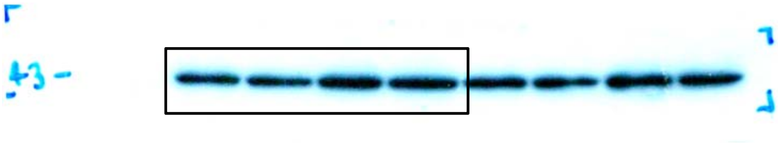

DR5 (55 kDa):

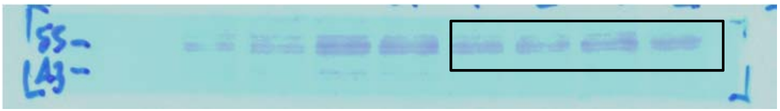

β-actin (43 kDa):

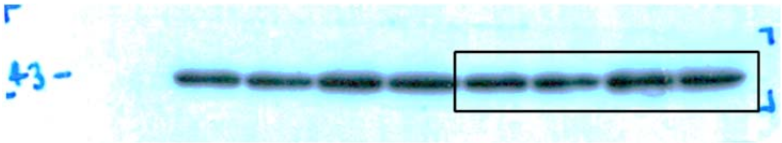

Supplement figure 7

Original blot figure (Figure 4C) (INS-1)

Bax (23 kDa):

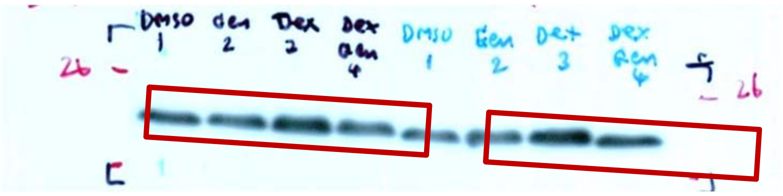

β-actin (43 kDa):

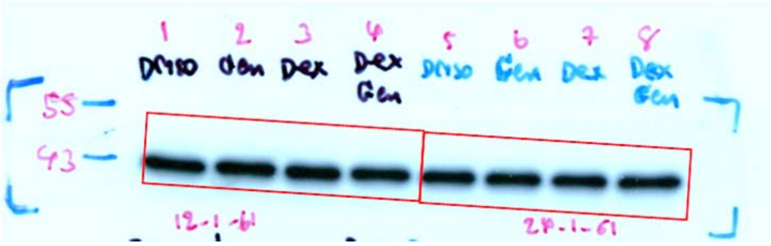

Bax (23 kDa):

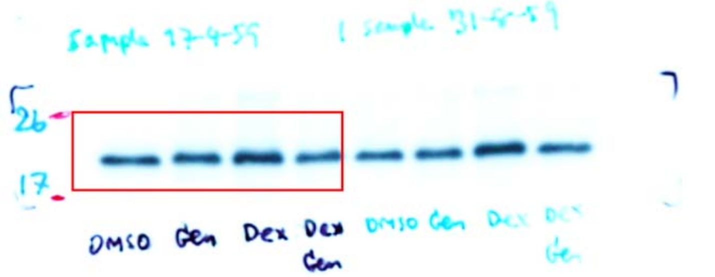

β-actin (43 kDa):

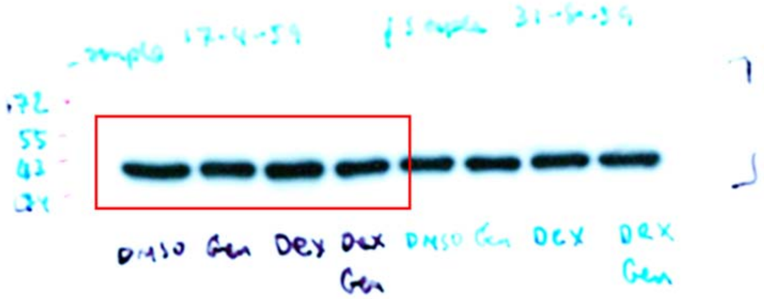

Supplement figure 8

Original blot figure (Figure 4D) (INS-1)

Bcl2 (26 kDa):

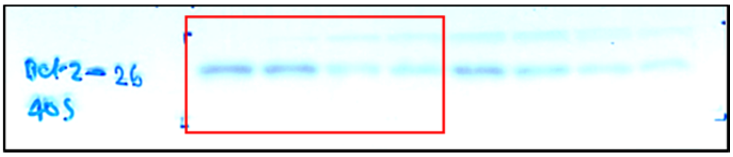

β-actin (43 kDa):

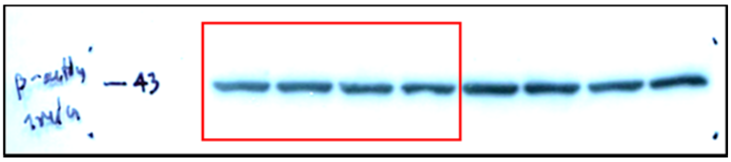

Bcl2 (26 kDa):

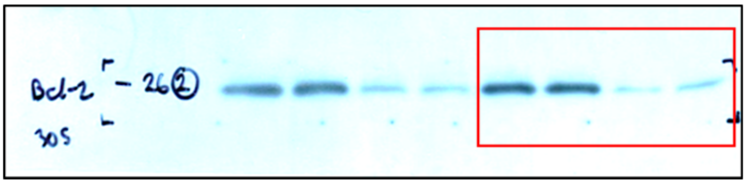

β-actin (43 kDa):

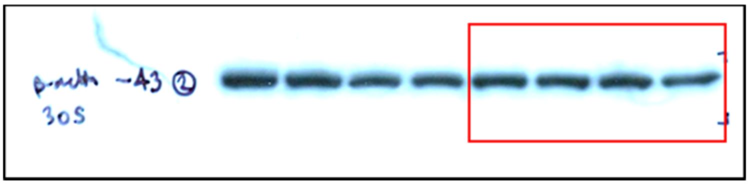

Bcl2 (26 kDa):

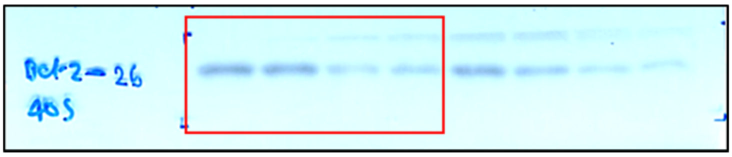

β-actin (43 kDa):

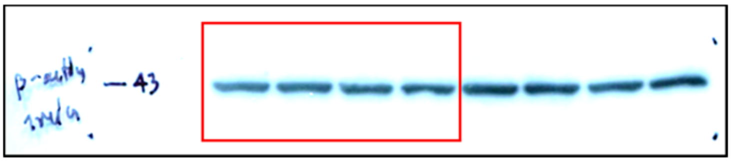

Supplement figure 9

Original blot figure (Figure 5) (INS-1)

pNF-κB (65 kDa):

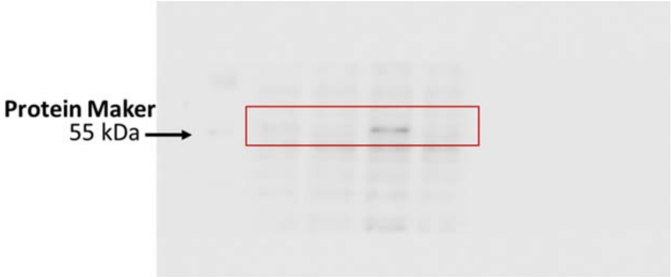

β-actin (43 kDa):

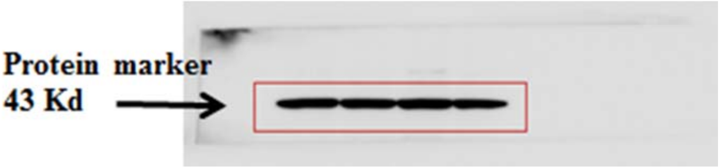

pNF-κB (65 kDa):

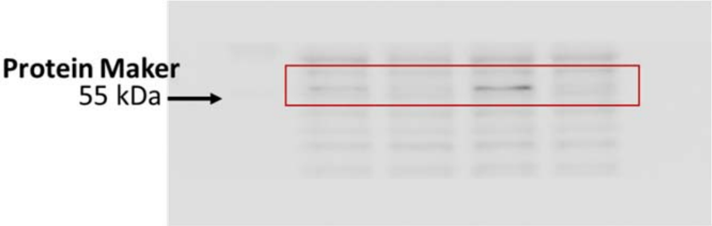

β-actin (43 kDa):

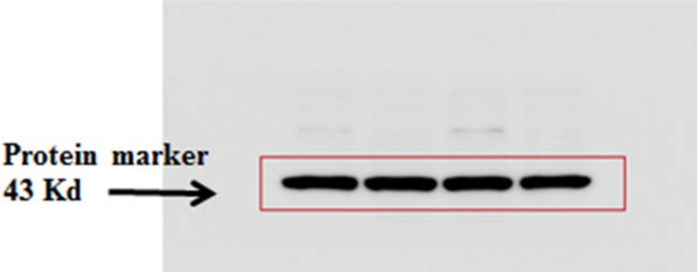

pNF-κB (65 kDa):

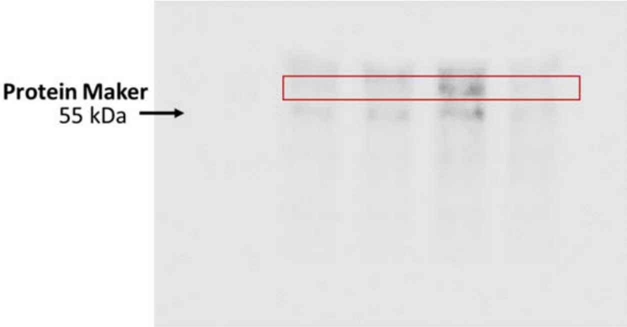

β-actin (43 kDa):

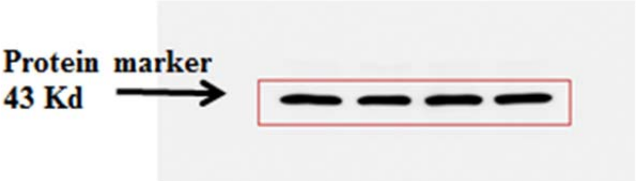

Supplement figure 10
